# Evidence of symptom profiles consistent with posttraumatic stress disorder and complex posttraumatic stress disorder in different trauma samples

**DOI:** 10.3402/ejpt.v5.24221

**Published:** 2014-05-19

**Authors:** Ask Elklit, Philip Hyland, Mark Shevlin

**Affiliations:** 1 National Centre for Psychotraumatology, Institute of Psychology, University of Southern Denmark, Odense, Denmark; 2 Psychology Research Institute, School of Psychology, Faculty of Life and Health Sciences, University of Ulster, Londonderry, United Kingdom; 3 National College of Ireland, Dublin, Ireland

**Keywords:** PTSD, complex PTSD, trauma, ICD-11, latent class analysis

## Abstract

**Background:**

The *International Classification of Diseases, 11th version* (ICD-11), proposes two related stress and trauma-related disorders, posttraumatic stress disorder (PTSD) and complex PTSD (CPTSD). A diagnosis of CPTSD requires that in addition to the PTSD symptoms, an individual must also endorse symptoms in three major domains: (1) affective dysregulation, (2) negative self-concepts, and (3) interpersonal problems. This study aimed to determine if the naturally occurring distribution of symptoms in three groups of traumatised individuals (bereavement, sexual victimisation, and physical assault) were consistent with the ICD-11, PTSD, and CPTSD specification. The study also investigated whether these groups differed on a range of other psychological problems.

**Methods and Results:**

Participants completed self-report measures of each symptom group and latent class analyses consistently found that a three class solution was best. The classes were “PTSD only,” “CPTSD,” and “low PTSD/CPTSD.” These classes differed significantly on measures of depression, anxiety, dissociation, sleep disturbances, somatisation, interpersonal sensitivity, and aggression. The “CPTSD” class in the three samples scored highest on all the variables, with the “PTSD only” class scoring lower and the “low PTSD/CPTSD” class the lowest.

**Conclusion:**

This study provides evidence to support the diagnostic structure of CPTSD and indicted that CPTSD is associated with a broad range of other psychological problems.

Posttraumatic stress disorder (PTSD) as a clinical condition has been the subject of intense empirical investigation in recent decades. A large proportion of this research has focused on identifying the most accurate and parsimonious conceptualisation of the disorder which would ultimately serve to guide diagnosis (Elhai & Palmieri, 2011; Yufik & Simms, 2010). Much of this work was spurred by the *Diagnostic and Statistical Manual of Mental Disorders* (4th Ed.; *DSM–IV*; American Psychiatric Association, 1994), specifying that PTSD was best defined in terms of three clusters of symptoms: r-experiencing, avoidance, and arousal. However, nearly two decades of research consistently undermined this model of PTSD and instead suggested that PTSD was best conceptualised in terms of two alternative four-factor models: one model defined in terms of the presence of a large group of non-specific symptoms termed “dysphoria” (Simms, Watson, & Doebbeling, 2002), and another model defined in terms of the presence of dissociative symptoms termed “emotional numbing” (King, Leskin, King, & Weathers, 1998). A large body of confirmatory factor analytic studies consistently failed to identify a superior model (Yufik & Simms, 2010). Attempts have been made to reconcile these contradictory findings, with recent work suggesting that the dysphoria and emotional numbing models should not be considered as competing models of the same disorder, but rather that these models are reflective of two different sub-populations of individuals suffering from a stressor-related condition (Shevlin & Elklit, 2012). These results are suggestive of alternative forms of PTSD, and broadly indicated that the traditional three-factor model of PTSD was incorrect. Accordingly, the recently published DSM-5 (American Psychiatric Association, 2013) presented significant alternations to the conceptualisation of PTSD. The DSM-5 now defines PTSD in terms of four clusters of symptoms: re-experiencing, avoidance, negative alterations in mood and cognition, and hyperarousal. Two subtypes are also included: pre-school PTSD and dissociative PTSD.

DSM-5 has expanded the diagnostic nomenclature with the inclusion of three new symptoms related to affective dysregulation and negative self-concept, along with the specifier for two new subtypes. This reformed conceptualisation of PTSD is at odds with the notion of clinical utility which is of pressing concern given that international research has demonstrated that mental health clinicians tend to eschew subtype specifiers and prefer a limited number of symptoms to guide diagnoses (Reed, Correia, Esparza, Saxena, & Maj, 2011). Additionally, the current DSM-5 formulation is also problematic in a research context in light of the *National Institute of Mental Health's* (NIMH) recent announcement of suspension of all funding for research based on the DSM's formulation of mental health disorders.

An alternative conceptualisation of PTSD is presented in the *International Classification of Diseases, 11th version* (*ICD-11*), which is due to be completed in 2015. In contrast to the DMS-5, the ICD-11's guiding principle for classification development is clinical utility. As such the ICD-11 proposes two connected stress- and trauma-related disorders: PTSD and complex PTSD (CPTSD; Maercker et al., 2013). The experience of any kind of stressful life event is viewed as a “gateway” for a consideration of a diagnosis of PTSD or CPTSD. The symptom profile rather than the trauma history becomes the focus of the diagnostic effort. Rather than the 20 symptoms outlined in DSM-5, the ICD-11 includes six symptoms of PTSD which comprise three symptom clusters: (1) re-experiencing of the traumatic event(s) in the present accompanied by emotions of fear or horror; (2) avoidance of traumatic reminders; and (3) a sense of current threat that is manifested by excessive hypervigilance or an enhanced startle reaction. The inclusion of just six symptoms not only aids the clinical utility in terms of diagnosis, but serves to define PTSD as a fear-based disorder and thus distinguishes it from other psychiatric disorders. Such classification could likely lead to a reduction in the very high level of comorbidity observed between PTSD and other psychiatric disorders (e.g., depression) when a DSM framework is utilised (Kessler et al., 1995; Zlotnick et al., 2006).

The ICD-11 classification of CPTSD includes the PTSD formulation as a core component of a diagnosis, but in addition includes symptoms that affect the individual in three major domains: (1) affective dysregulation, (2) negative self-concepts, and (3) interpersonal problems. Collectively these have been described as disturbances in self-organisation (DSO). A diagnosis of CPTSD requires that in addition to the PTSD symptoms, an individual must display at least one symptom from each of these three clusters. Affective dysregulation includes many different symptoms such as violent outbursts, excessive crying, anhedonia, self-destructive behaviour, dissociation, or emotional numbing. A negative self-concept refers to the perception of a diminished or defeated sense of self that can arise from the experience of a trauma, and is characterised by the presence of persistent negative beliefs about oneself along with feelings of guilt and shame. Interpersonal problems are identified by an inability to build or maintain close and intimate personal bonds. There is evidence that chronic exposure to stressful life events can increases one's likelihood of reporting such DSO (Briere & Rickards, 2007) however it is important to note that the ICD-11 simply states that exposure to chronic stress increases an individual's probability of developing CPTSD rather than being a requirement for a diagnosis of CPTSD.

Given that this proposed reformulation of the diagnostic framework of PTSD is only due for publication in the ICD-11 in 2015, limited empirical findings exist with respect to the validity of such a distinction between PTSD and CPTSD. Cloitre, Garvert, Brewin, Bryant, and Maercker (2013) recently conducted a study among 302 people who had been exposed to both chronic traumatic life events and single-incident events. Utilising latent profile analytic techniques, the researchers found strong evidence in favour of the ICD-11's distinction between PTSD and CPTSD. Cloitre and colleagues identified three separate classes of trauma-exposed individuals. The largest class (36.1%) included those who reported elevated symptoms of PTSD, as well as elevated symptoms in all three domains of affective dysregulation, negative self-concepts, and interpersonal problems. This class represented those suffering from CPTSD. The second class (31.8%) was defined in terms of strong endorsement of all PTSD symptoms, but low levels of endorsement of the items relating to affective dysregulation, negative self-concepts, and interpersonal problems. This class represented those experiencing PTSD. The third and final class (32.1%) included those healthy individuals who had a low level of endorsement across all items.

Cloitre et al.'s (2013) results further indicated that those persons who reported experiencing chronic trauma-exposure (childhood abuse) were twice as likely to experience CPTSD as compared to PTSD. Alternatively, experiencing a single-incident traumatic life event (exposure to the 9/11 terrorist attacks in New York City) was associated with a four times greater likelihood of having PTSD as compared to CPTSD. These results indicate that repeated exposure to traumatic life events is a risk factor for CPTSD, however it is important to note that a substantial percentage (25%) of those individuals who experienced chronic-stressors developed PTSD rather than CPTSD, and a similar number of people (12%) who experienced a single-incident trauma developed CPTSD rather than PTSD. Cloitre et al.'s analysis also demonstrated importantly that the PTSD and CPTSD groups did not differ in terms of severity of PTSD symptoms, and that CPTSD was associated with greater functional impairment than was PTSD.

These results provide initial evidence for the validity of the ICD-11's proposed classification of two distinct disorders. However, given that the study of Cloitre et al. (2013) was the first such study to assess for the presence of two distinct constructs in a trauma-exposed sample, significantly more research is required in order to determine the accuracy of a distinction between PTSD and CPTSD, as proposed by the ICD-11. The current study seeks to extend the findings of Cloitre and colleagues by testing for the presence of both PTSD and CPTSD within a diverse group of traumatised people using latent class analysis (LCA). LCA is a statistical method used to identify homogeneous groups (or classes) from categorical multivariate data. In the present study, LCA was used (based on PTSD and DSO symptoms) to determine if there was evidence of groups of participants, or classes, that matched the profile of PTSD and CPTSD in three different trauma groups.

This current study has two main aims. The first aim is to test the hypothesis that for each of the three samples utilised in the current analysis there would be three distinct groups of participants, or classes. We hypothesised that one class would display a high probability of endorsing the PTSD symptoms and the symptoms relating to affective dysregulation, negative self-concepts, and interpersonal problems (*CPTSD class*); a second class would display a high probability of endorsing items relating to PTSD and a low probability of endorsing items relating to affective dysregulation, negative self-concepts, and interpersonal problems (*PTSD class*); and a third class would display a low probability of endorsing any of the symptoms of PTSD or CPTSD (*low PTSD/CPTSD*). The second aim was to investigate differences in impairment across the resultant classes. It was hypothesised that the CPTSD and PTSD classes would score higher than the low PTSD/CPTSD class on a range of measures of psychological problems, and that the CPTSD class would score higher than the PTSD class on all measures.

## Method

### Participants

Data from 1,251 participants were used from three independent samples that consisted of (1) bereaved parents after the death of a child (*N*=607), (2) rape victims (*N*=449), and (3) victims of physical assault (*N*=214). Across all three samples the mean age was 29.43 years (SD=9.96) with 65.1% of the total sample being female.

Sample 1 consisted of 607 parents who had lost a child to infant death. The mean age was 33.99 years (SD=5.85) with 350 (57.7%) of the sample being female. Most parents were members of the Danish “National Association of Infant Death” and experienced the loss of a child on average 3.3 years from the time of participating in the study. Participants were recruited via a postal questionnaire (response rate was 46%) or through invitation to participate when attending the maternity ward or neonatal department of two large Danish hospitals. The study was approved by the Aarhus University Institutional Review Board and the Danish National Association of Infant Death.

Sample 2 consisted of 430 victims of sexual trauma. The sample was predominantly female (97.7%) with a mean age of 29.43 years (SD=9.69). The participants had all contacted the Centre for Rape Victims (CRV) located within the University Hospital of Aarhus (Denmark) and were recruited by invitation when attending the CRV. This study received ethical approval from the Aarhus University Institutional Review Board and the CRV.

Sample 3 consisted of 214 victims of physical assault. The sample had a mean age of 30.2 (SD 12.29) and 73.4% were male. They were recruited during a 1 month period from an emergency ward at the University Hospital of Aarhus. During registration potential participants were offered the opportunity to take part in the study if the primary inclusion criterion was met, that they experienced “grievous bodily harm caused by another person.” Within 2 weeks all consenting (and over 18 years old) participants were sent questionnaires and the response rate was 35.4%. This study was approved by the Danish Authority for Registers and by the regional Helsinki Committee.

### Measures

Twelve items that comprised the PTSD/CPTSD item set were selected from two standardised measures, the Trauma Symptom Checklist (TSC; Briere & Runtz, 1989) and the Harvard Trauma Questionnaire Part IV (HTQ: Mollica et al., 1992). The items representing PTSD and CPTSD are shown in Table 1.

**Table 1:** Items representing PTSD and CPTSD

Factor	Cluster	Test items
PTSD	Re-experiencing	HTQ 3. Recurrent nightmares
		HTQ 2. Feeling as though the event is happening again
	Avoidance	HTQ 15. Avoiding thought or feelings associated with the traumatic or hurtful events
		HTQ 11. Avoiding activities that remind you of the traumatic or hurtful event
	Sense of threat	HTQ 9. Feeling on guard
		HTQ 6. Being jumpy or easily startled
Complex PTSD	Affect dysregulation	TSC 16. Temper outburst that you could not control
		TSC 14. Crying easily
	Negative self-concept	TSC 28. Feelings of inferiority or insecurity
		TSC 29. Blaming yourself
	Interpersonal problems	TSC 6. Feeling isolated from other people
		HTQ 27. Feeling that you have no one to rely upon

Both measures used a four-point Likert response scale. The TSC asks participants to rate the frequency of occurrence (*How often have you experienced each of the following in the last month?*) of each symptom on a scale ranging from “Never” (0) and “Often” (3). The HTQ asks participants to rate the distress each symptom has caused them in the previous week (*Decide how much the symptoms bothered you in the last week*) on a scale ranging from “Not at all” (1) to “Extremely” (4). The item scores were recoded into binary variable scores and a symptom was rated as present if the item corresponding to the symptom was scored 2 or greater on the TSC items, and 3 or greater for the HTQ items.

The original TSC contained 33 items, and Elklit (1990) added two more items and these are used to compute seven subscales: depression, anxiety, dissociation, sleep disturbances, somatisation, interpersonal sensitivity, and aggression. Sample questions are shown in Table 2.

**Table 2:** Example items from the Trauma Symptom Checklist subscales

Item	Subscale
Low sex drive	Depression
Uncontrollable crying	
Feelings of inferiority	Anxiety
Having trouble breathing	
Feeling that things are “unreal”	Dissociation
“Spacing out”—going away in your mind	
Restless sleep	Sleep disturbances
Waking up early in the morning and can't get back to sleep	
Headaches	Somatisation
Stomach problems	
Trouble getting along with others	Interpersonal sensitivity
Loneliness	
Trouble controlling temper	Aggression
Desire to hurt others physically	

Five items from the TSC were used in the PTSD/CPTSD item set so they were not included in the subscales (3 removed from depression, 1 from interpersonal sensitivity, and 1 from aggression). The correlations between the mean scores of the original subscales and the shortened subscale scores were high (depression *r*=0.97, *p*<0.01; interpersonal sensitivity *r*=1.00, *p*<0.01; aggression *r*=0.95, *p*<0.01). Estimates of reliability for each of the subscales for all participants were generally high: depression (*α*=0.81), anxiety (*α*=0.83), dissociation (*α*=0.84), sleep disturbances (*α*=0.86), somatisation (*α*=0.84), interpersonal sensitivity (*α*=0.73), and aggression (*α*=0.62). The low reliability of the aggression subscale may be because it is comprised of only three items. The overall reliability for the TSC was also high (*α*=0.95).

### Analysis

For each sample, a series of LCA models, with one through to six classes, were specified and estimated using Mplus 7.00 (Muthén & Muthén, 2012). All models were based on responses to the 12 binary HTQ and TSC items and estimated using robust maximum likelihood (Yuan & Bentler, 2000). To avoid solutions based on local maxima 500 random sets of starting values were used initially with 100 final stage optimisations. Relative model fit was compared using information theory based fit statistics; Akaike Information Criterion (AIC; Akaike, 1987), Bayesian Information Criterion (BIC; Schwartz, 1978) and sample-size-adjusted BIC (ssaBIC; Sclove, 1987). The model that produces the lowest values can be judged the best model. The Lo–Mendell–Rubin adjusted likelihood ratio test (LMRA-LRT: Lo, Mendell, & Rubin, 2001) and the bootstrapped likelihood ratio test were also used to compare models where a non-significant value indicates that the model with one less class should be accepted. Evidence from simulation studies have indicated that the BIC was the best information criterion for identifying the correct number of classes (Nylund, Asparouhov, & Muthén, 2007). Each model also included the seven TSC subscales as distal outcomes. Mplus provides an overall test of association, and pairwise class comparisons, using Wald chi-square test. This provides a test of difference between the unconstrained and constrained model where the means are specified to be equal. This approach protects the overall type 1 error rate.

## Results

The frequencies and percentages of symptom endorsement for PTSD and DSO symptoms are presented in Table 3. There was a significant association between the level of symptom endorsement and trauma type. In general the participants from the sexual trauma group had the highest level of symptom endorsement across all PTSD and DSO symptoms, whereas the bereaved parents group tended to have higher levels of endorsement compared to the assault group.

**Table 3:** Frequencies and percentages of symptom endorsement for PTSD and DSO symptoms

Group	HTQ 2	HTQ 3	HTQ 15	HTQ 11	HTQ 9	HTQ 6	TSC 16	TSC 14	TSC 28	TSC 29	TSC 6	HTQ 27
Bereaved parents	108	39	54	76	202	174	69	63	43	47	38	57
	33.5%	15.8%	13.5%	19.7%	33.5%	32.4%	27.9%	26.0%	22.3%	21.7%	25.9%	19.8%
Physical assault	66	42	71	80	135	85	34	32	26	30	24	49
	20.5%	17.0%	17.8%	20.7%	22.4%	15.8%	13.8%	13.2%	13.5%	13.8%	16.3%	17.0%
Sexual trauma	148	166	275	230	266	278	144	147	124	140	85	182
	46.0%	67.2%	68.8%	59.6%	44.1%	51.8%	58.3%	60.7%	64.2%	64.5%	57.8%	63.2%
Total	322	247	400	386	603	537	247	242	193	217	147	288
χ^2^	34.98	152.98	330.91	194.09	91.87	114.36	75.15	87.88	89.19	107.34	42.35	142.87
df	2	2	2	2	2	2	2	2	2	2	2	2
P	<.01	<.01	<.01	<.01	<.01	<.01	<.01	<.01	<.01	<.01	<.01	<.01

Note: HTQ items were endorsed if a response was 3 or greater; TSC items were endorsed if a response was 2 or greater.

**Fig. 1:**
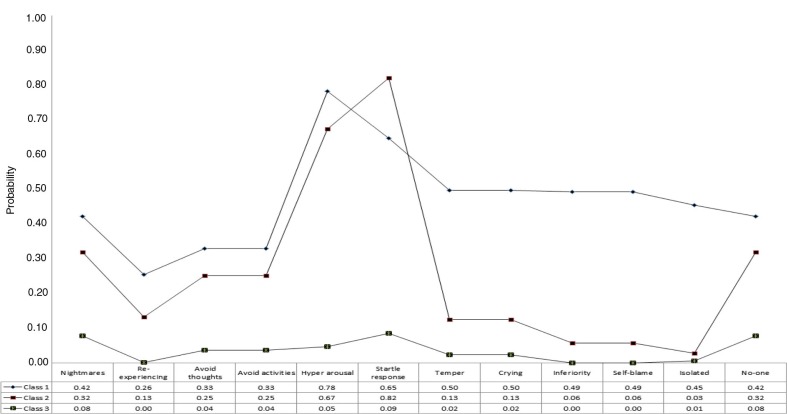
Profile plot of estimates from latent class analysis of complex PTSD symptoms: sudden infant death sample.

The fit statistics for the LCA analyses based on the bereaved parents’ data are reported in Table 4. The BIC has the smallest value for a solution with three classes and the LMRA-LRT becomes non-significant for the four class solution, therefore the three class solution was considered the best. Figure 1 shows the profile plot for the three class solution. Class 1 (*N*=63, 10.4%) was the smallest and was characterised by a high probability of endorsement of PTSD and DSO symptoms. This class was labelled the “CPTSD” class. Class 2 (*N*=152, 25.2%) had a similar profile of probabilities for the PTSD symptoms as the CPTSD class, but the endorsement probabilities for the DSO symptoms were markedly lower. This class was labelled the “PTSD” class. The third class was the largest (*N*=389, 64.4%) and the probabilities of endorsement were low for all symptoms, and this class was labelled the “low PTSD/CPTSD” class.

**Table 4:** Fit statistics for latent class analysis of CPTSD symptoms: bereaved parents sample

Classes	Loglikelihood	AIC	BIC	ssaBIC	LMRA-LRT	BS LRT
1	−2645.088	5314.176	5367.019	5328.922		
2	−2282.240	4614.481	4724.570	4645.201	717.082**	725.696**
**3**	−**2218.991**	**4513.983**	**4681.318**	**4560.678**	124.996**	126.498**
4	−2192.378	4486.756	4711.339	4549.426	52.595	53.226**
5	−2175.759	4479.518	4761.347	4558.163	32.844*	33.238
6	−2162.437	4478.874	4817.950	4573.494	26.327	26.644

Note: Statistical significance:

** *p*<.0005

* *p*<.05. AIC=Akaike information criterion, BIC=Bayesian information criterion, ssaBIC=sample-size-adjusted BIC, LMRA-LRT=Lo–Mendell–Rubin adjusted likelihood ratio test. Best model in bold.

**Fig. 2:**
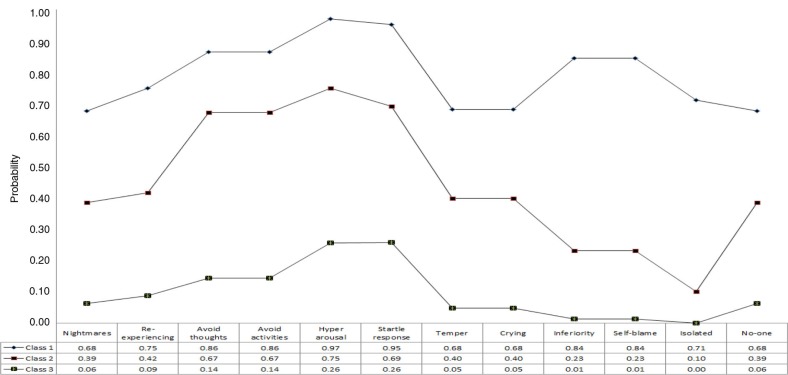
Profile plot of estimates from latent class analysis of complex PTSD symptoms: sexual trauma sample.

The fit statistics for the LCA analyses based on the sexual trauma victims data are reported in Table 5. The BIC (and the ssaBIC) has the smallest value for a solution with three classes and the LMRA-LRT becomes non-significant for the four class solution, therefore the three class solution was considered the best. Figure 2 shows the profile plot for the three class solution. Class 1 (*N*=93, 20.7%) was the smallest and was characterised by a high probability of endorsement of PTSD and DSO symptoms. This class was labelled the “CPTSD” class. Class 2 (*N*=194, 43.2%) had a similar profile of probabilities for the PTSD and Affect dysregulation symptoms as the CPTSD class, but the endorsement probabilities for the remaining DSO symptoms were markedly lower. This class was labelled the “PTSD” class and was the largest class. The third class (*N*=162, 36.1%) and the probabilities of endorsement were low for all symptoms. This was labelled the “low PTSD/CPTSD” class.

**Table 5:** Fit Statistics for latent class analysis of complex PTSD symptoms: sexual trauma sample

Classes	Loglikelihood	AIC	BIC	ssaBIC	LMRA- LRT	BS LRT
1	−3381.803	6787.607	6836.891	6798.807		
2	−2888.374	5826.747	5929.423	5850.082	974.584**	986.859**
**3**	−**2819.851**	**5715.702**	**5871.769**	**5751.171**	135.341**	137.045**
4	−2802.246	5706.493	5915.951	5754.097	34.771	35.209*
5	−2787.336	5702.672	5965.522	5762.411	29.450	29.821
6	−2773.165	5700.331	6016.571	5772.203	27.989	28.341

Note: Statistical significance:

** *p*<.0005

* *p*<.05. AIC=Akaike information criterion, BIC=Bayesian information criterion, ssaBIC=sample-size-adjusted BIC, LMRA-LRT=Lo–Mendell–Rubin adjusted likelihood ratio test. Best model in bold.

**Fig. 3:**
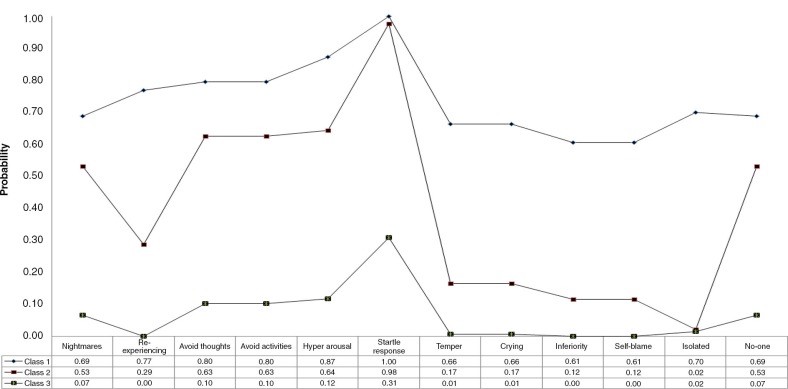
Profile plot of estimates from latent class analysis of complex PTSD symptoms: physical assault sample.

The fit statistics for the LCA analyses based on the physical assault victim's data are reported in Table 6. The BIC has the smallest value for a solution with three classes; however the LMRA-LRT was non-significant for this solution also. The LMRA-LRT probability value for the three class solution was only marginally greater than 0.05 so this solution was considered the best. Figure 3 shows the profile plot. Class 1 was the smallest (*N*=28, 13.0%) and the probabilities of endorsement for all symptoms was high. This was labelled “CPTSD” class. Class 2 (*N*=72, 33.6%) had high probabilities for the PTSD symptoms compared to the DSO symptoms; the symptoms relating to exaggerated startle response and lack of some-one to rely on were relatively high, and for re-experiencing the probability was low. This class was labelled the “PTSD” class. The third class (*N*=114, 53.4%) was the largest and was characterised by a low probability of endorsement of PTSD and DSO symptoms with the exception of the exaggerated startle response symptom. This class was labelled “low PTSD/CPTSD.”

**Table 6:** Fit statistics for latent class analysis of complex PTSD symptoms: physical assault sample

Classes	Loglikelihood	AIC	BIC	ssaBIC	LMRA-LRT	BS LRT
1	−1331.473	2686.946	2727.338	2689.313		
2	−1081.286	2212.573	2296.722	2217.504	493.301**	500.373**
**3**	−**1044.629**	**2165.257**	**2293.164**	**2172.752**	**72.280**	73.316**
4	−1026.864	2155.728	2327.393	2165.786	35.027	35.529**
5	−1013.742	2155.483	2370.906	2168.106	25.873	26.244
6	−999.597	2153.194	2412.374	2168.380	27.650	28.047

Note: Statistical significance:

** *p*<.0005. AIC=Akaike information criterion, BIC=Bayesian information criterion, ssaBIC=sample-size-adjusted BIC, LMRA-LRT=Lo–Mendell–Rubin adjusted likelihood ratio test. Best model in bold.

There were significant differences between all three classes on all TSC subscales for all samples. In addition all pairwise comparisons were statistically significant. The pattern of results was similar across samples; without exception the CPTSD classes scored significantly higher than the PTSD classes, and the PTSD classes scored higher than the low PTSD/CPTSD classes. The means and tests of mean differences are reported in Tables 7–9 .

**Table 7:** Tests of differences of means of TSC subscales across classes: bereaved parents

	Depression	Anxiety	Dissociation	Sleep disturbance	Somatisation	Interpersonal sensitivity	Aggression
Class 1: CPTSD	2.11 (.06)	1.82 (.06)	1.90 (.07)	2.00 (.09)	1.98 (.07)	2.07 (.06)	1.66 (.06)
Class 2: PTSD	1.63 (.03)	1.44 (.03)	1.42 (.03)	1.58 (.05)	1.49 (.04)	1.49 (.03)	1.30 (.02)
Class 3: Low PTSD/CPTSD	1.39 (.02)	1.17 (.01)	1.20 (.01)	1.29 (.02)	1.24 (.01)	1.32 (.01)	1.16 (.01)
Overall test^ (Wald *χ* ^2^)	208.70*	180.04*	155.12*	93.69*	147.52*	175.54*	106.28*
Pairwise tests^^ (Wald *χ* ^2^)							
Class 1 vs. 2	38.14*	59.67*	41.69*	26.87*	37.61*	27.52*	26.21*
Class 1 vs. 3	49.44*	28.51*	38.02*	14.00*	32.38*	67.80*	31.00*
Class 2 vs. 3	150.64*	104.88*	100.84*	55.29*	93.86*	133.77*	72.46*

Note:

^^ all tests df=2

^ all tests df=1. Statistical significance

* *p*<.01.

**Table 8:** Tests of differences of means of TSC subscales across classes: sexual trauma victims

	Depression	Anxiety	Dissociation	Sleep disturbance	Somatisation	Interpersonal sensitivity	Aggression
Class 1: CPTSD	2.82 (.06)	2.59 (.07)	2.65 (.07)	2.93 (.08)	2.60 (.07)	2.39 (.70)	2.28 (.08)
Class 2: PTSD	2.14 (.04)	1.88 (.04)	1.92 (.04)	2.20 (.06)	1.88 (.04)	1.89 (.04)	1.78 (.05)
Class 3: Low PTSD/CPTSD	1.47 (.03)	1.31 (.03)	1.36 (.03)	1.50 (.05)	1.32 (.03)	1.40 (.03)	1.26 (.03)
Overall test^ (Wald *χ* ^2^)	259.83*	239.48*	218.65*	162.06*	183.81*	163.69*	118.10*
Pairwise tests^^ (Wald *χ* ^2^)							
Class 1 vs. 2	77.88*	85.33*	74.55*	46.04*	69.14*	36.98*	25.94*
Class 1 vs. 3	362.89*	318.65*	282.93*	217.48*	259.42*	180.39*	135.40*
Class 2 vs. 3	163.05*	148.64*	122.72*	77.20*	108.00*	90.53*	87.24*

Note:

^^ all tests df=2

^ all tests df=1. Statistical significance

* *p*<.01.

**Table 9:** Tests of differences of means of TSC subscales across classes: physical assault victims

	Depression	Anxiety	Dissociation	Sleep disturbance	Somatisation	Interpersonal sensitivity	Aggression
Class 1: CPTSD	2.71 (.12)	2.55 (.11)	2.57 (.15)	2.88 (.15)	2.66 (.12)	2.34 (.11)	2.11 (.13)
Class 2: PTSD	1.85 (.07)	1.77 (.06)	1.62 (.06)	1.97 (.09)	1.79 (.07)	1.64 (.05)	1.49 (.05)
Class 3: Low PTSD/CPTSD	1.32 (.04)	1.27 (.03)	1.25 (.03)	1.32 (.05)	1.30 (.04)	1.38 (.03)	1.23 (.03)
Overall test^^ (Wald *χ* ^2^)	70.53*	91.90*	43.38*	61.53*	72.11*	50.03*	35.17*
Pairwise tests^ (Wald *χ* ^2^)							
Class 1 vs. 2	40.00*	52.40*	28.88*	37.17*	34.20*	17.47*	35.17*
Class 1 vs. 3	111.54*	124.75*	66.51*	89.54*	108.78*	72.26*	45.30*
Class 2 vs. 3	36.32*	38.06*	30.21*	23.77*	36.21*	35.09*	20.27*

Note:

^^ all tests df=2

^ all tests df=1. Statistical significance

* *p*<.01.

## Discussion

The primary objective of the current study was to investigate the proposed ICD-11 distinction between two traumatic stress disorders; PTSD and CPTSD. The first study carried out (Cloitre et al., 2013) to investigate the presence of these distinct disorders was supportive. The current study sought to substantially further assess the validity of the proposed ICD-11 distinction between PTSD and CPTSD in three distinct trauma groups. In contrast to the latent profile analysis conducted by Cloitre and colleagues, the current study utilised a categorical analysis of symptom endorsement and therefore LCA was performed to investigate our working hypothesis that three distinct classes would be identified in each sample of trauma victims: a class consistent with a PTSD diagnosis, a class consistent with a CPTSD diagnosis, and a class that was low on all symptoms. In the samples of bereaved parents, sexual assault victims, and victims of physical assault, the results of the LCA clearly identified three discrete classes consistent with this prediction.

Results of the LCA were informative in that they indicated that trauma history was an important risk factor for the type of diagnosis received. Sexual trauma victims were the most likely group of individuals to report CPTSD (20.7%), followed by physical assault victims (13%), and bereaved parents (10.4%). In terms of the development of PTSD only, victims of sexual trauma were at the highest risk (43.2%), followed by physical assault victims (33.6%), and bereaved parents (25.2%). It should also be noted that in each sample of trauma victims a substantial proportion of individuals were highly resilient and demonstrated very low symptoms of PTSD or CPTSD. Sixty-four per cent of the bereaved parents were in the low PTSD/CPTSD class, 53% of the physical assault victims were in the low PTSD/CPTSD class, and 36% of rape victims were in the low PTSD/CPTSD class. These results are informative for clinical practice as they offer an indication that trauma history can be a guiding factor in making a differential diagnosis between PTSD and CPTSD; however, as with the results of Cloitre et al. (2013), current results provide strong evidence that one's trauma history is not a determining factor in the type of diagnosis one will receive.

Results of the current study were also broadly supportive of our second hypothesis that individuals reporting symptoms of CPTSD would demonstrate the highest level of functional impairment. Results indicated that for all measures of psychopathology (depression, anxiety, dissociation, sleep disturbances, somatisation, interpersonal sensitivity, and aggression) the CPTSD class was significantly more impaired than the PTSD class and the low PTSD/CPTSD class. Additionally, the PTSD class demonstrated significantly higher levels of impairment across all domains compared to the low PTSD/CPTSD class.

Overall these results are in line with the proposal of the ICD-11 to classify two distinct traumatic conditions distinguished on the basis of symptom presentation rather than trauma history. Moreover, current results suggest that a greatly reduced set of indicators of PTSD in the ICD-11 (6) as compared to the DSM-5 (20) is an effective method of identifying this disorder and thus consistent with the ICD-11's guiding principle of clinical utility. This distinction between PTSD and CPTSD is also of great clinical importance given the observation that those individuals in the CPTSD class exhibited significantly higher levels of impairment across all seven measures of psychological distress.

In addition to PTSD and CPTSD, the ICD-11 also proposes the inclusion of another stress-response syndrome; prolonged grief disorder (PGD). As outlined by Maercker and Lalor (2012), PGD and PTSD share many commonalities. Specifically, the core symptoms of PGD relate to yearning symptoms and avoidance/emotional numbing symptoms, which are consistent with the intrusive symptoms of PTSD and the affective dysregulation symptoms of CPTSD, respectively. This is important to consider in light of the results of the current study as the sample of bereaved parents may well be better classified as experiencing PGD rather than PTSD or CPTSD. The bereaved parents were the least likely of each sample to belong to either the CPTSD class or the PTSD class, and a higher percentage belonged to the low PTSD/CPTSD class than the sexual and physical assault victims, respectively. Current results may indicate a degree of symptom relatedness between PGD and both PTSD and CPTSD, therefore subsequent research will be required to improve understanding of the discriminating factors between these related conditions.

Findings of the current study must necessarily be considered in light of a number of limitations. First, participants for two of the three samples utilised in the current study were recruited from the Danish population, therefore it is unknown whether current results will generalise to other populations. Second, the analysis for the current study was based on the use of self-report measures not specifically designed to capture the ICD-11 classifications of PTSD and CPTSD. The development and validation of both self-report questionnaires and clinical-interviews consistent with ICD-11 guidelines are necessary for a more robust test to take place. Third, the indicators used to measure the DSO symptoms are best-guess items from measures available and may not be most representative of each DSO category. Development of uniform and reliable clinical and self-report measures for ICD-11 is an important next step.

In conclusion, results of the current study among three unique groups of trauma victims provide evidence in support of the distinction between two related traumatic stress disorders (PTSD and CPTSD), as proposed in the upcoming ICD-11. This study demonstrates that trauma history is a risk factor rather than a determining factor in the type of traumatic stress response one may exhibit; and finally, that those individuals who develop CPTSD subsequent to exposure to a traumatic life event, experience substantially greater psychological and functional impairment than those individuals with PTSD.
